# Editorial: Tick and Tick-Borne Pathogens: Molecular and Immune Targets for Control Strategies

**DOI:** 10.3389/fphys.2020.00744

**Published:** 2020-08-07

**Authors:** Abid Ali, Albert Mulenga, Itabajara Silva Vaz

**Affiliations:** ^1^Department of Zoology, Abdul Wali Khan University Mardan, Mardan, Pakistan; ^2^Department of Veterinary Pathobiology, College of Veterinary Medicine, Texas A&M University, College Station, TX, United States; ^3^Centro de Biotecnologia, Universidade Federal do Rio Grande do Sul, Porto Alegre, Brazil; ^4^Faculdade de Veterinaria, Universidade Federal do Rio Grande do Sul, Porto Alegre, Brazil

**Keywords:** tick, tick-borne pathogens, control, molecular-targets, immune-targets

Ticks are obligate hematophagous ectoparasites of domestic animals, humans, and wildlife. Ticks can be found in areas around the world ranging from the Arctic to tropical regions, and are known for their negative impact. They are capable of transmitting a wide range of pathogens including protozoa, viruses, and bacteria including spirochetes and rickettsia. The resulting diseases can potentially cause major production losses in livestock, thereby reducing farming incomes, increasing cost to consumers, and threatening trade between regions and/or world markets. Climate change has an impact on the distribution of ticks and tick-borne pathogens because tick species select a set of ecological conditions and biotopes that determine their geographic distributions and outline risk areas for their associated pathogens' transmission. In particular, arthropod vectors such as ticks, are vulnerable to these climatic changes as their population, survival, and development depend on factor like vegetation, availability of a host, photoperiod, moister and climatic conditions. Tick density, distribution, and their capability of pathogen transmission are thus effected (Ali et al.; Gerardi et al.).

Novel approaches have emerged, and the limitations of present operational protocols have been reduced to improve and standardize the laboratory procedures to lower costs, and to obtain a better understanding of tick and tick-borne pathogen interactions. Convenient and low-cost techniques provide a great opportunity to identify new targets for the future control of TBVs (Talactac et al.). Almazán et al. showed that sheep inoculated with tick cells infected with the *Anaplasma phagocytophilum* developed an infection 4 days post-inoculation (dpi) and the infected nymphs of *Ixodes ricinus* were able to transmit the *A. phagocytophilum* to naive infested sheep. The sheep transmitted the bacteria to 2.7% nymphs engorged as larvae during persistent infection. Among several other tick-borne diseases, tropical theileriosis caused by *Theileria annulata* infection is a significant livestock disease—especially in crossbreed cattle. The current study highlights the genetic and allelic diversity present among the Indian *T. annulata* parasites and its vaccine using a microsatellite marker, *tams1* sequencing, and GBS. The findings indicate that a heterogeneous parasitic population is prevalent in India, causing theileriosis, which may render the vaccine ineffective due to their high diversity. Parasite diversity data will be helpful in revamping new vaccines to control the disease (Roy et al.).

The hemocytes of *Rhipicephalus microplus* females, after *Metarhizium robertsii* infection associated with the cytotoxicity, were evaluated. Cytoplasmic vacuoles were observed in hemocytes of infected female ticks with electron densities, and in lipid droplets in close contact to low electron density vacuoles, as well as the formation of autophagosomes and subcellular material in different stages of degradation. This study reported fungal cytotoxicity, analyzing ultrastructural effects on hemocytes of *R. microplus* infected with entomopathogenic fungi (Fiorotti et al.). The genes that are required by tick-borne pathogens, such as *A. phagocytophilum* for host invasion and proliferation, were studied. Among them, several genes were found to be upregulated within human and tick cell lines (HL-60 and ISE6). Genes with unknown functions were found to play a disproportionate role in the establishment of infection (Nelson et al.).

The effects of an experimental infection with *Rickettsia rickettsii* on the global gene expression profile of the *A. aureolatum* salivary gland (SG), was determined by next-generation RNA sequencing. A total of 260 coding sequences (CDSs) were modulated by infection, among which 161 were upregulated and 99 were downregulated. Regarding CDSs in the immunity category, one sequence encoding one microplusin-like antimicrobial peptide (AMP) was chosen because, when there is a *R. rickettsii* infection, microplusin is significantly upregulated in both the salivary glands and the midgut tissues of *A. aureolatum*. The expression of microplusin was significantly upregulated in the SG as well as in the midgut (MG) of infected *A. aureolatum*. The knockdown of microplusin expression by RNA interference caused a significant increase in the prevalence of infected ticks (Martins et al.).

The porin functions in *Haemaphysalis longicornis* blood feeding and *Babesia* infection, and the relationship between porin and porin-related apoptosis genes such as B-cell lymphoma (Bcl), cytochrome complex (Cytc), caspase 2 (Cas2), and caspase 8 (Cas8), was analyzed. Porin expression levels were higher in the infected vs. uninfected nymphs during blood feeding, except at 1-day-partially-fed and 0 to 1-day post-engorgement. The highest *B. microti* burden negatively affected porin mRNA levels in both nymphs and female adults. Porin knockdown affected body weight and *Babesia* infection levels and significantly downregulated the expression levels of Cytc and Bcl in *H. longicornis* female ticks. Finally, the obtained results suggest that *H. longicornis* porin might interfere with blood feeding and *B. microti* infection (Zheng et al.).

A large amount of genomic and proteomic data of different organisms are available in public data banks. The genomic and proteomic sequence information of pathogens can provide an aid in detection and characterization of the novel therapeutic targets and vaccine candidates. The regulome (transcription factors-target genes interactions) plays a critical role in the cell's response to pathogen infection. The application of regulomics to tick-pathogen interactions will advance the understanding of these molecular interactions and contribute to the identification of novel control targets, to control tick and tick-borne diseases. In this article, *in silico* approaches were applied to model how *A. phagocytophilum* infection modulates the tick vector regulome. This proof-of-concept study provided support for the use of a network analysis in the study of the regulome response to infection, resulting in new information on tick-pathogen interactions and potential targets for developing interventions, to control tick infestations and pathogen transmission (Artigas-Jerónimo, Villar et al.).

A first comprehensive report of tick fauna was published from a northern state of Pakistan, which revealed that tick species comprising of six genera, were infesting diverse hosts including humans. These tick species included *R. microplus, Hyalomma anatolicum, Argas persicus, H. impeltatum, R. turanicus, R. haemaphysaloides, R. annulatus, Haemaphysalis montgomeryi, H. indica*, and *H. punctate*. *H. marginatum, R. sanguineus*, and *H. longicornis, A. gervaisi, A. exornatum, A. latum, Dermacentor marginatus*. It is important to mention that *H. punctata, Amb*. *exornatum*, and *Amblyomma latum* correspond to imported tick populations (authors personal communication). The phylogenetic analysis of tick *R. microplus*, based on partial mitochondrial cytochrome oxidase subunit I (COX1), 16S ribosomal RNA (16S rRNA), and internal transcribed spacer 2 (ITS2) sequences, revealed that *R. microplus* prevalent in this region belong to clade C, which include ticks originating from Bangladesh, Malaysia, and India (Ali et al.).

Blood feeding by ticks requires prolonged contact with a host's tissue and blood, and it has been suggested that the co-evolution of ticks with their natural hosts has resulted in the selection of an appropriate set of salivary components that allow ticks to evade both specific and non-specific host immunity in order to successfully obtain blood (Bullard et al.). In their quest for a blood meal, ticks transmit pathogens and inject a cocktail of bioactive molecules into their vertebrate hosts. Tick-borne pathogens have developed mechanisms to survive in the arthropod vector by manipulating gene expression, based on the midgut or salivary environment (Gerardi et al.; Antunes et al.).

Although not a replacement for the whole organism, research on tick cell lines provides a foundation for studies at less expensive *in vitro*, an alternative to *in vivo* tick feeding experiments (Al-Rofaai and Bell-Sakyi). Acaricides are the main components of integrated tick control strategies—despite their limited success and the associated hazards. It is under this scenario that anti-tick vaccines have emerged as an alternative tool for tick control. The potential advantages of vaccine-based control strategies include: cost-effectiveness, avoidance of environmental contamination, prevention of drug-resistance, possible prevention of pathogen transmission, and its potential applicability in a wide variety of hosts. A comprehensive understanding of tick proteins and their physiological roles can help shed light on how these parasites overcome host defenses, revealing new molecules of potential use for tick control and therapeutic applications (Artigas-Jerónimo, Villar et al.). The identification of conserved proteins across various parasites or vectors may provide the opportunity to develop a viable universal vaccine for control of multiple arthropods infestations and a platform for further characterization of how their evolution can meet species-specific demands. For instance, the conserved functional evolution of subolesin/akirin correlates with the protective capacity shown by these proteins in vaccine formulations for the control of different arthropod and pathogen species (Artigas-Jerónimo, Villar et al.).

Components of the tick salivary glands have attracted attention as candidate antigens for anti-tick vaccines. Knowledge about the role of tick salivary proteins in tick physiology is important for a better understanding of the host-parasite relationships, drug targets, and the search for a novel candidate antigen for vaccine development. Moreover, tick salivary molecules have been observed to potentially exert cytotoxic and cytolytic effects on various tumor cells that have anti-angiogenic properties. Therefore, research has been focused on the identification of major tick salivary protein families exploitable in medical applications such as immunomodulation, inhibition of hemostasis, and inflammation, and the potential, opportunities, and challenges in searching for novel tick-derived drugs have been discussed (Chmelar et al.; Štibrániová et al.). A review article described that the tick feeding site may be considered as a closed system and could be treated as an ideal equilibrium system. Several tick proteins may have functional relevant concentrations and affinities at the feeding site. The feeding site is not an ideal closed system, but leads to a possible overestimation of tick protein concentration at the feeding site, and consequently, an overestimation of functional relevance (Mans).

A kinin precursor sequence was predicted from several tick species, and all species showed an expansion of kinin paracopies. The “kinin core” (minimal active sequence) of tick kinins, FX1X2WGamide, is similar to insect sequences. A library of 14 small molecules, antagonists of mammalian neurokinin (NK) receptors, was screened using this endpoint assay and antagonists of NK receptors displayed selectivity (>10,000-fold) on the tick kinin receptor. Future approaches may accelerate kinin discoveries such as the identification of molecules for acaricide development (Xiong et al.).

Complete mitochondrial genomes of the bisexual and parthenogenetic populations were analyzed, and the expression of the mitochondrial protein-coding genes was evaluated. Single nucleotide polymorphism analysis showed that ~200 bases were different, and the phylogenetic tree showed that the genetic distance between bisexual and parthenogenetic populations was lower than the subspecies. The expressions of the mitochondrial protein-coding genes, at different feeding stages of the bisexual and parthenogenetic population, revealed differences in expression patterns, which suggest that they might trigger specific energy utilization mechanisms due to their different reproductive strategies (Wang et al.).

Resistance to tick infestation by a vertebrate host reduces the chances of pathogen transmission (Lew-Tabor et al., 2017). Demonstrations of cellular and molecular mechanism underlying tick-resistance is also important for the development of an anti-tick vaccine. In a review article, the history on tick-resistance overviewed the recent findings, particularly the role of basophils in the tick-resistance manifestation. Basophil accumulation has been observed at the tick re-infestation site, even though the frequency of basophils among cellular infiltrates varies in different animal species. Skin-resident memory CD4+ T cells contribute to the recruitment of basophils to the tick re-infestation site through production of IL-3 in mice. Depletion of basophils before the tick re-infestation abolishes tick-resistance, demonstrating the crucial role of basophils in tick-resistance. The activation of basophils via IgE and its receptor FcεRI is essential, and histamine released from activated basophils functions as an important effector molecule in murine tick-resistance. First, tick infestation triggers the production of IgE against saliva antigens in the host, and blood-circulating basophils bind such IgE on the cell surface via FcεRI. In the second infestation, IgE-armed basophils are recruited to the tick-feeding sites and are activated by tick saliva antigens to release histamine that promotes epidermal hyperplasia, contributing to tick resistance (Karasuyama et al.).

Various approaches have been developed to obtain an effective vaccine against *R. microplus* and other ticks. The glycoprotein BM86, a tick gut epithelial cell protein, became the first commercial vaccine against tick infestation. However, this vaccine demonstrates quite variable efficacy and does not confer enough protection against several tick populations. Developing an anti-tick vaccine consisting of one or more common tick antigens, capable of triggering protective immune responses against heterologous tick challenges, would be economically and technically attractive. Additionally, elucidating the role of tick molecules may be a promising avenue for new approaches to developing therapeutic applications (Artigas-Jerónimo, Estrada-Peña et al.). A Vaccinomics based study, presented in an original research article, showed that recombinant ferritin 2 from *I. ricinus* (IrFER2) and tick protein extract (TPE) consisting of soluble proteins from the internal organs of partially fed *I. ricinus* females, conferred a strong immune response in calves and significantly reduced the feeding success of both nymphs and adults (Knorr et al.).

It has been suggested that a cocktail of candidate antigens might be more effective for the development of an effective tick vaccine (Parizi et al., 2012). Two *I. ricinus* proteins, heme lipoprotein and an uncharacterized secreted protein, and five *Dermacentor reticulatus* proteins, a glypican-like protein, secreted protein involved in homophilic cell adhesion, sulfate/anion exchanger, signal peptidase complex subunit 3, and uncharacterized secreted protein proteins, were identified as the most effective protective antigens resulting in vaccines that affect multiple tick developmental stages. The combination of some of these antigens might increase vaccine efficacy through antigen synergy for the control of tick infestations and may potentially affect pathogen infection and transmission (Contreras et al.).

Interfering in tick reproduction is an interesting target for the development of new methods for tick control, since ticks have fast reproduction rates and a large number of descendants. To maintain the tick embryo development during incubation, all supplements are derived from maternally derived nutrients. Therefore, the deposition of compounds in the egg occurs during egg formation and determine the success of embryo development and hatching. Since embryogenesis is a metabolically intensive process, the energy supplementation is vital for a perfect embryo development. The insulin signaling pathway regulates glucose homeostasis and protein kinase B (PKB, or AKT), glycogen synthase kinase 3 (GSK-3), and target of rapamycin (TOR) are key enzymes of this signaling pathway. Waltero et al. studied the characterization of the TOR and two other downstream effectors, S6 kinase (S6K) and eukaryotic translation initiation factor 4E-binding protein 1 (4E-BP1), to determine the role of this enzyme in tick reproduction. Additionally, with the goal of identifying a useful target, Michael III et al. focused a detailed review on the knowledge of the molecular structure and functional role of the tick vitellogenin receptor. They related that inhibition of the expression of the receptor is possible to infer oocyte maturation, egg deposition, and pathogen transmission.

## Author Contributions

All authors listed have made a substantial, direct and intellectual contribution to the work, and approved it for publication.

## Conflict of Interest

The authors declare that the research was conducted in the absence of any commercial or financial relationships that could be construed as a potential conflict of interest.
